# Inequalities in the prevalence of undiagnosed hypertension among Bangladeshi adults: evidence from a nationwide survey

**DOI:** 10.1186/s12939-019-0930-5

**Published:** 2019-02-15

**Authors:** Sayem Ahmed, Md. Tariqujjaman, Md. Arafat Rahman, Md. Zahid Hasan, Md. Mehedi Hasan

**Affiliations:** 1 International Centre for Diarrhoeal Disease Research, Bangladesh (icddr,b), Dhaka, 1212 Bangladesh; 2 Department of Learning, Informatics, Management and Ethics, Karolinska Institutet, Stockholm, 171 77 Sweden; 3 Department of Tropical Disease Biology, Liverpool School of Tropical Medicine (LSTM), Liverpool, L3 5QA UK; 4 Department of Economics, Faculty of Business and Economics, Macquarie University, Sydney, NSW 2109 Australia; 5 Institute for Social Science Research, The University of Queensland, Indooroopilly 4068 Queensland, Australia

**Keywords:** Undiagnosed hypertension, Non-communicable disease, Concentration index, Bangladesh

## Abstract

**Background:**

In recent years, developing countries like Bangladesh are facing a higher burden of non-communicable diseases such as hypertension as a result of demographic transition. Prevalence of hypertension is often studied in this setting. However, evidence on undiagnosed hypertension is not widely available in the existing literature. Therefore, the current study focuses on inequalities in the prevalence of undiagnosed hypertension in Bangladesh.

**Methods:**

A total of 8835 participants aged 35+ years were included in this study using nationally representative Bangladesh Demographic and Health Survey 2011 (BDHS). In the survey, systolic blood pressure (SBP) and diastolic blood pressure (DBP) of these participants were measured three times with approximately 10 minutes of an interval between each measurement. Any respondent with either SBP ≥ 140 mmHg or DBP ≥ 90 mmHg was considered as patient with hypertension as per the guidelines from American Heart Association. Among the participants, undiagnosed hypertension was defined as having SBP > =140 mmHg or DBP > =90 mmHg and never taking prescribed medicine or being told by health professionals to lower/control blood pressure. Multiple logistic regression analysis was applied for identifying factors associated with undiagnosed hypertension. Further, socioeconomic inequalities in the prevalence of undiagnosed hypertension were estimated using Concentration Index (C).

**Results:**

We found 978 (59.9% of the total) were undiagnosed among 1685 hypertensive patients studied. Regression analysis showed individuals with being underweight, having poor socioeconomic conditions, and lower educational qualifications were more likely to have undiagnosed hypertension. A similar association between undiagnosed hypertension and socioeconomic quintiles was observed using concentration index (C = − 0.07). On the other hand, individuals from higher age group (50–64 or above), female sex, and Sylhet region were at lower risk of undiagnosed hypertension.

**Conclusions:**

This study showed that a large proportion of the cases with hypertension are remained undiagnosed in Bangladesh, especially among the poor and low educated population. Screening and awareness building initiatives on hypertension should be taken for this group of population to reduce the burden of undiagnosed hypertension.

## Introduction

Hypertension is a leading risk factor for cardiovascular diseases, stroke, kidney failure, disability, and premature death [1, 2]. Globally, in 2008, about 40% (aged 25 years or above) had been diagnosed with hypertension and currently, over one billion people are living with this condition [3]. In the Asian region, hypertension has become a major public health challenge, affecting more than 35% of the adult population [4]. Due to demographic transition, the number of elderly people is increasing in low-and-middle-income countries (LMICs) which is leading to a higher prevalence of non-communicable diseases (NCDs) like hypertension in this region [5, 6]. It was estimated that more than 13% of the total deaths around the world were related to hypertension in 2010. Such hypertension related mortality is rising rapidly in LMICs [7]. In the South-East Asian region, hypertension affects one in three adults and the trend of hypertension is increasing [8]. The prevalence of hypertension among all adults and elderly people (age ≥ 60 years) in Bangladesh is 25 and 40% respectively [9].

In LMICs, like Bangladesh, hypertension disproportionately affects many people and is often undiagnosed compared to high-income countries [3]. For prevention, treatment, and control of hypertension, it is crucial to diagnose this condition. Moreover, this undiagnosed hypertensive condition may lead to serious health complications or even deaths.

The Government of Bangladesh is constitutionally committed to providing minimum healthcare to every citizen that includes health services, health education, health promotion, and rehabilitation. The health systems of this country have 3 tiers structure including primary (e.g. Community Clinic, Upazila Health Complex), secondary (e.g. District Hospital), and tertiary level (e.g. Medical College Hospital, Specialized Facilities) healthcare facilities covering all villages, sub-districts, and districts. The secondary and tertiary level of healthcare facilities are currently delivering treatment for hypertension and the government has planned to extend this service up to primary level [10]. Beside the public facilities, there are a number of private and non-profit NGO clinics, hospitals, and diagnostic centres across the country providing healthcare services.

Several studies estimated the prevalence of hypertension in Bangladesh [5, 11, 12]. However, the evidence on the national prevalence of undiagnosed hypertension is still limited. Islam et al. 2016 and Khanam et al. 2012 estimated prevalence of undiagnosed hypertension in rural settings using a small sample size [9, 13]. Islam et al. 2016 found that 82% among the hypertensive patients were undiagnosed in a rural district [13] and Khanam et al. 2012 showed that 11.1% of the total population were undiagnosed for hypertension from the Matlab rural surveillance sites of Bangladesh [9]. The national level estimate of undiagnosed hypertension prevalence is essential for planning effective strategies to reduce the burden of hypertension. Therefore, we aimed to estimate the prevalence of undiagnosed hypertension and its socioeconomic inequalities in Bangladesh.

## Methods

### Design and settings

For this study, we used secondary data derived from the Bangladesh Demographic and Health Survey (BDHS) 2011. BDHS employed two-stage stratified sampling procedure to collect the data. In the first stage, BDHS selected 600 (207 in urban and 393 in rural) enumeration areas with probability proportional to size. In the second stage, on average a systematic sample of 30 households was selected per enumeration areas.

### Participants

**Fig. 1:**
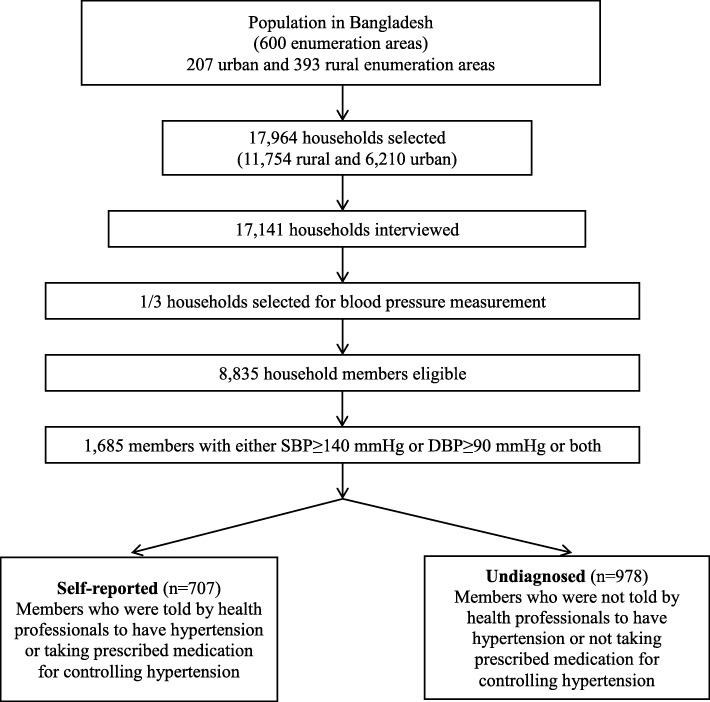
Sample selection flow chart. SBP = Systolic Blood Pressure, DBP = Diastolic Blood Pressure

In total 17,141 households consisting 83,731 individuals were surveyed. We included 8835 adult participants of both sexes aged 35+ years in this study since this group is at higher risk of hypertension [14]. We found 1685 cases with hypertension; systolic blood pressure (SBP) > =140 mmHg or diastolic blood pressure (DBP) > =90 mmHg. A detail of sample selection is presented in Fig. 1.

### Outcome variable

In BDHS 2011, the blood pressure level of participants was recorded and used for measuring hypertension. To measure blood pressure level “LIFE SOURCE UA-767 Plus Blood Pressure Monitor” device was used as recommended by World Health Organization (WHO). Well-trained health technicians were employed to measure and record the blood pressure of the participants. As the blood pressure levels vary within a short period of time, BDHS measured SBP and DBP three times with approximately 10 minutes of an interval between each measurement. Finally, the average of the last two measurements was considered to detect hypertension among the participants. Hypertension was defined using the guidelines from the American Heart Association (AHA) [14]. According to the AHA, a participant with SBP > =140 mmHg or DBP > =90 mmHg is diagnosed as a hypertension case. Among these cases with hypertension, undiagnosed hypertension was defined as having SBP > =140 mmHg or DBP > =90 mmHg and never taking prescribed medicine or being told by health professionals to lower/control blood pressure [15].

### Explanatory variables

The socioeconomic status of each household was measured by asset index constructed using principal component analysis (PCA) approach. The PCA is a widely used technique for computing asset indices based on the ownership of durable assets in the households and infrastructure and housing characteristics (e.g. source of water, sanitation facility, housing structure) [16]. Traditionally the technique was applied to continuous variables, however, Filmer and Pritchett (2001) argued that PCA can be a valid method for categorical and binary data like ownership of assets [17]. Higher scores of the index indicate more affluent households. All the households were ranked from the lowest to the highest asset score and divided into 5 quintiles. Confounders included in this study were age group (35–49 years, 50–64 years, and 65+ years), sex (Male and Female), educational status (No institutional education, Primary, Secondary, and Higher), marital status (Currently married, Divorced/widowed/separated), nutritional status (Underweight: body-mass index (BMI) < 18.5 kg/m^2^, Normal: BMI > =18.5 kg/m^2^ and BMI < 25 kg/m^2^, and Overweight/Obese: BMI > =25 kg/m^2^), households with children (dummy: 0 = No child and 1 = has child), administrative division (Barisal, Chittagong, Dhaka, Khulna, Rajshahi, Rangpur, and Sylhet), and place of residence (Urban and Rural).

### Statistical analyses

Univariate analysis for calculating percentages of categorical variables was applied to describe the characteristics of the sampled population. Bivariate analysis using Chi-square test was employed to investigate the differentials in the prevalence of undiagnosed hypertension over background characteristics. All the analyses were done taking into account the complex survey design for capturing variations due to weighting and designing of the survey.

Multiple logistic regression models were applied to explore the potential determinants of undiagnosed hypertension. In multiple logistic regression models, we included only the variables those were found significantly associated with undiagnosed hypertension in the simple logistic regression models. The results of simple and multiple logistic regression analyses were presented in terms of unadjusted odds ratios (OR) and adjusted odds ratios (AOR) along with their respective 95% confidence intervals (CIs). Three separate multiple logistic regression models were fitted at three levels of background characteristics namely, individual level (model 2), household level (model 3), and finally community level (model 4).

The concentration index (C), along with standard error (SE) of C, was estimated to show the direction of undiagnosed hypertension prevalence across different socioeconomic groups of households. In calculating C, we ranked the households according to their socioeconomic characteristics from the poorest to the richest. Distribution of undiagnosed hypertension was measured by plotting a concentration curve representing the cumulative proportion of undiagnosed hypertension in Y-axis and cumulative proportions of the population in X-axis. If the prevalence of undiagnosed hypertension is equally distributed across socioeconomic groups, the concentration curve will coincide with the diagonal. In contrast, if there are inequalities in the prevalence of undiagnosed hypertension, the concentration curve will deviate from the diagonal. The C is defined as twice the area between the concentration curve and the diagonal [18–20]. The index value can range between − 1 and + 1, a positive value implies prevalence of undiagnosed hypertension is more concentrated among the better-off socioeconomic group and a negative value implies prevalence is more concentrated among less affluent group [19, 21]. STATA (version 13) was used to perform all the analyses [22].

## Results

### Characteristics of the study participants

The background characteristics of the study participants are presented in Table 1. Among the patients studied, more than one-third (37.8%) were between 35 and 49 years of age and 61.7% were female. Around half (51.0%) of the patients had no institutional education whereas only 9.0% had higher education. However, almost two out of every three patients were currently married (73.8%). Around 57.3% of the patients had normal BMI and 71.3% lived in rural area.

**Table 1:** Characteristics of study population

Characteristics	Frequency (*N* = 1685)	Percentage (%)	95% Confidence interval	
			Lower bound (%)	Upper bound (%)
Individual characteristics				
Age in years				
35–49	635	37.8	35.4	40.3
50–64	575	33.8	31.3	36.3
> =65	475	28.4	25.8	31.0
Sex				
Male	663	38.3	35.8	40.7
Female	1022	61.7	59.3	64.2
Education				
No education	804	51.0	48.1	53.9
Primary	411	23.5	21.0	25.9
Secondary	305	16.5	14.6	18.5
Higher	165	9.0	7.3	10.7
Marital status				
Currently married	1244	73.8	71.4	76.1
Divorced/separated/others	441	26.2	23.9	28.6
Body weight				
Normal	899	57.3	54.7	59.9
Underweight	341	21.7	19.3	24.0
Overweight/obese	366	21.0	18.8	23.2
Household characteristics				
HH having child				
No child	1070	64.1	61.3	66.9
Has child	615	35.9	33.1	38.7
Wealth index				
Poorest	232	15.2	12.9	18.0
Poorer	257	16.6	14.3	18.9
Middle	286	17.4	15.1	19.7
Richer	375	22.0	19.5	24.6
Richest	535	28.7	25.7	31.8
Community characteristics				
Division				
Barisal	180	5.6	4.8	6.5
Chittagong	192	12.5	10.8	14.2
Dhaka	304	34.0	31.1	36.9
Khulna	327	16.7	14.4	19.0
Rajshahi	225	12.5	10.8	14.2
Rangpur	302	14.5	12.8	16.2
Sylhet	155	4.1	3.3	4.9
Place of residence				
Urban	649	28.7	26.2	31.2
Rural	1036	71.3	68.8	73.8

### Prevalence of undiagnosed hypertension

Among 1685 hypertensive patients (SBP > =140 or DBP > =90) studied, 978 cases with hypertension (59.9% of total) were found to be undiagnosed. About 65.9% of these undiagnosed patients were aged between 35 and 49 years. The prevalence of undiagnosed hypertension was higher among male patients (66.9%) compared to female (55.5%) and it was lower among the higher education group (49.4%) compared to no education group (63.9%). Moreover, the rate of undiagnosed hypertension was found to be higher among the underweight people (65.5%). In terms of wealth index, the prevalence of undiagnosed hypertension was higher among the people from the poorest (69.7%) and poorer (69.6%) quintiles compared to people belonged to the richest (50.8%) quintile. Significant variations in the prevalence of undiagnosed hypertension were observed among people from seven administrative divisions (Chi-square value = 22.1, *p*-value< 0.01) (Table 2).

**Table 2:** Prevalence of undiagnosed hypertension among patients aged 35 years or above by background characteristics

Characteristics	Undiagnosed Hypertension (%)	95% Confidence interval		Chi-square value (*p*-value)
		Lower bound (%)	Upper bound (%)	
Individual characteristics				
Age in years				15.2 (0.002)
35–49	65.9	61.9	69.7	
50–64	56.1	51.0	61.0	
> =65	56.5	51.4	61.5	
Sex				21.5 (<0.001)
Male	66.9	62.8	70.8	
Female	55.5	51.8	59.2	
Education				14.8 (0.007)
No education	63.9	59.9	67.7	
Primary	57.5	51.9	62.9	
Secondary	56.7	50.4	62.8	
Higher	49.4	41.4	57.4	
Marital status				3.9 (0.085)
Currently married	61.3	58.2	64.3	
Divorced/separated/others	56.0	50.3	61.4	
Body weight				31.9 (<0.001)
Normal	62.6	59.0	66.1	
Underweight	65.5	59.1	71.4	
Overweight/obese	46.7	40.6	52.9	
Household characteristics				
HH having child				0.0 (0.861)
No child	59.7	56.2	63.1	
Has child	60.2	55.7	64.5	
Wealth index				40.5 (<0.001)
Poorest	69.7	63.0	75.7	
Poorer	69.6	62.4	76.0	
Middle	61.8	55.5	67.7	
Richer	56.1	50.3	61.7	
Richest	50.8	45.9	55.8	
Community characteristics				
Division				22.1 (0.004)
Khulna	61.6	55.6	67.4	
Barisal	55.9	47.8	63.7	
Chittagong	52.9	45.6	60.1	
Dhaka	59.7	53.8	65.2	
Rajshahi	58.7	51.6	65.5	
Rangpur	70.7	64.3	76.4	
Sylhet	47.0	36.3	58.0	
Place of residence				4.5 (0.063)
Urban	55.9	50.8	60.8	
Rural	61.5	58.2	64.8	
Total	59.9	57.1	62.6	–

### Determinants of undiagnosed hypertension

In the unadjusted logistic regression model, we found that patient’s age, education, BMI, administrative division, place of residence, and wealth status were significantly associated with undiagnosed hypertension. Results from multiple logistic regression models, adjusted for individual characteristics (model 2) and that of household characteristics, (model 3) were similar to the unadjusted model. The logistic regression model was adjusted by all the significant variables (significantly associated with undiagnosed hypertension) in the unadjusted model.

In model 4, old aged patients (age 50 years or more) were less likely to have undiagnosed hypertension compared to the younger aged (age 35–49 years) patients (AOR = 0.53; 95% CI = 0.41–0.68 for 50–64 years and AOR = 0.49; 95% CI = 0.36–0.66 for 65+ years). Patients who received secondary and higher education were 30% (AOR = 0.70; 95% CI = 0.5–0.98) and 57% (AOR = 0.43; 95% CI = 0.27–0.68) less likely to have undiagnosed hypertension respectively as compared to the patients with no education. The likelihood of having undiagnosed hypertension were less among female patients (AOR = 0.68; 95% CI = 0.48–0.97) and overweight/obese patients (AOR = 0.65; 95% CI = 0.5–0.85) compared to male and patients with normal body weight respectively. Patients from the poorest and poorer asset quintiles were 1.86 (AOR = 1.86; 95% CI = 1.25–2.78) and 1.78 (AOR = 1.78; 95% CI = 1.16–2.72) times more likely to have undiagnosed hypertension than the patients from the richest wealth quintile. Compared to the Khulna division, people living in Sylhet division were 45% less likely to have undiagnosed hypertension (AOR = 0.55; 95% CI = 0.37–0.84) (Table 3).

**Table 3:** Risk factors associated with undiagnosed hypertension among patients aged 35 years or more in Bangladesh

Characteristics	Odds Ratio (95% Confidence interval)			
	Model 1 (univariate)^1^	Model 2^2^	Model 3^3^	Model 4^4^
Individual characteristics				
Age in years				
35–49	1.00	1.00	1.00	1.00
50–64	0.66*** (0.53–0.84)	0.51*** (0.4–0.66)	0.52*** (0.41–0.68)	0.53*** (0.41–0.68)
> =65	0.69*** (0.54–0.88)	0.46*** (0.34–0.61)	0.47*** (0.35–0.64)	0.49*** (0.36–0.66)
Sex				
Male	1.00	1.00	1.00	1.00
Female	0.65*** (0.53–0.8)	0.68** (0.48–0.96)	0.68** (0.48–0.97)	0.68** (0.48–0.97)
Education				
No education	1.00	1.00	1.00	1.00
Primary	0.81* (0.63–1.03)	0.71** (0.54–0.93)	0.79* (0.6–1.04)	0.81 (0.61–1.07)
Secondary	0.76** (0.58–0.99)	0.56*** (0.41–0.76)	0.70** (0.5–0.97)	0.70** (0.5–0.98)
Higher	0.52*** (0.37–0.73)	0.34*** (0.22–0.52)	0.44*** (0.28–0.69)	0.43*** (0.27–0.68)
Marital status				
Currently married	1.00	–	–	–
Divorced/separated/others	0.83* (0.67–1.03)	–	–	–
Body mass index				
Normal	1.00	1.00	1.00	1.00
Underweight	1.23 (0.95–1.59)	1.35** (1.03–1.78)	1.25 (0.94–1.65)	1.28* (0.96–1.7)
Overweight/obese	0.55*** (0.43–0.71)	0.60*** (0.46–0.78)	0.65*** (0.49–0.84)	0.65*** (0.5–0.85)
Household characteristics				
HH having child				
No child	1.00	–	–	–
Has child	0.92 (0.75–1.12)	–	–	–
Wealth index				
Richest	1.00	–	1.00	1.00
Richer	1.33** (1.02–1.74)	–	1.15 (0.86–1.54)	1.11 (0.82–1.5)
Middle	1.59*** (1.19–2.12)	–	1.28 (0.92–1.79)	1.25 (0.87–1.8)
Poorer	2.53*** (1.84–3.47)	–	1.90*** (1.32–2.74)	1.86*** (1.25–2.78)
Poorest	2.61*** (1.88–3.63)	–	1.83*** (1.23–2.72)	1.78*** (1.16–2.72)
Community characteristics				
Division				
Khulna	1.00	–	–	1.00
Barisal	0.82 (0.57–1.18)	–	–	0.8 (0.53–1.19)
Chittagong	0.71* (0.5–1.02)	–	–	0.88 (0.6–1.3)
Dhaka	0.97 (0.71–1.33)	–	–	1.03 (0.73–1.45)
Rajshahi	0.93 (0.66–1.32)	–	–	0.97 (0.67–1.41)
Rangpur	1.35* (0.97–1.87)			1.1 (0.78–1.56)
Sylhet	0.56*** (0.38–0.83)	–	–	0.55*** (0.37–0.84)
Place of residence				
Urban	1.00	–	–	1.00
Rural	1.40*** (1.15–1.71)	–	–	0.99 (0.77–1.28)

Note: *** denotes *p*-value< 0.01, ** denotes *p*-value< 0.05, * denotes *p*-value< 0.10

^1^Univariate logistic regression models considered each variable separately

^2^Model 2 considered only individual characteristics

^3^Model 3 considered individual and household characteristics

^4^Model 4 considered individual, household and community characteristics

### Socioeconomic inequalities in undiagnosed hypertension

Socioeconomic inequalities in the prevalence of undiagnosed hypertension are presented in Table 4. Findings showed that the prevalence of undiagnosed hypertension was distributed among poor socioeconomic groups (C = − 0.07; SE of C = 0.01). The absolute measure of inequality depicted that the prevalence of undiagnosed hypertension was greater by 18.9% (Q1-Q5 = 18.9%) among the poorest group than the richest group. Similarly, from the distribution of the prevalence of undiagnosed hypertension, we found poor (Q1) vs rich (Q5) ratio as 1.37 in Bangladesh. The disparities in undiagnosed hypertension was higher in Sylhet division (C = − 0.17, SE of C = 0.05) and lower in Dhaka (C = − 0.04, SE of C = 0.03) and Rajshahi (C = − 0.04, SE of C = 0.03) division.

**Table 4:** Socioeconomic inequalities in undiagnosed hypertension in Bangladesh

Characteristics	Poorest (Q1) (%)	Richest (Q5) (%)	Q1-Q5	Q1:Q5	Concentration index (C)	Standard error (SE)
Total	69.7	50.8	18.9	1.37	− 0.07	0.01
Individual characteristics						
Age in years						
35–49	78.9	56.6	22.3	1.39	−0.07	0.02
50–64	66.7	44.6	22.1	1.49	−0.10	0.02
> =65	64.7	48.3	16.4	1.34	−0.06	0.02
Sex						
Male	83.6	57.0	26.7	1.47	−0.08	0.02
Female	62.6	46.3	16.3	1.35	−0.07	0.02
Education						
No education	68.6	54.2	14.4	1.27	−0.05	0.02
Primary	75.7	46.6	29.0	1.62	−0.08	0.02
Secondary	77.3	52.5	24.8	1.47	−0.06	0.03
Higher	0.0	48.2	−48.2	0.00	−0.03	0.04
Marital status						
Currently married	72.4	52.4	20.0	1.38	−0.07	0.01
Divorced/separated/others	64.0	44.6	19.4	1.44	−0.07	0.02
Body weight						
Normal	76.6	57.1	19.5	1.34	−0.06	0.01
Underweight	66.7	60.9	5.8	1.10	−0.03	0.02
Overweight/obese	36.6	39.6	−3.1	0.92	−0.07	0.03
Household characteristics						
HH having child						
No child	69.6	49.5	20.2	1.41	−0.08	0.01
Has child	69.9	53.1	16.8	1.32	−0.05	0.02
Community characteristics						
Division						
Barisal	67.8	38.1	29.7	1.78	−0.09	0.04
Chittagong	59.3	39.4	19.9	1.51	−0.12	0.04
Dhaka	63.4	55.7	7.7	1.14	−0.04	0.03
Khulna	70.1	52.1	18.0	1.35	−0.07	0.02
Rajshahi	83.7	48.1	35.6	1.74	−0.04	0.03
Rangpur	77.3	58.4	18.9	1.32	−0.07	0.02
Sylhet	65.4	34.9	30.6	1.88	−0.17	0.05
Place of residence						
Urban	74.7	52.6	22.1	1.42	−0.04	0.02
Rural	69.3	47.6	21.8	1.46	−0.08	0.01

## Discussion

This study investigated the prevalence of undiagnosed hypertension among Bangladeshi adults and associated socioeconomic inequalities. It was observed that 59.9% remained hypertensive during the survey and was not diagnosed before. This could be due to lack of awareness, inaccessibility to screening services and the patient’s unwillingness to go to doctors for a regular check-up until any or related health complication arises [23]. Undiagnosed hypertension is highly prevalent in Bangladesh. Different estimates of undiagnosed hypertension were found in the literature. Islam et al. 2016 showed that among the hypertensive patients, the prevalence was 82% in the rural area [13]. Conversely, a much lower rate of undiagnosed hypertension was noticed by Khanam et al. 2012 in rural Bangladesh [9]. The variations could likely be due to the smaller sample domain, covering only rural participants from one to three sub-districts, differences in the age group of the selected participants. However, we selected participants aged 35+ years where Khanam et al. 2012 selected 30+ years for estimating the prevalence of undiagnosed hypertension [9].

We found the prevalence of undiagnosed hypertension was lower in higher educated participants compared to the lower educated participants. The possible reason could be the educated people are more aware of hypertension and have better health-seeking behaviour and also have relatively more affordability and accessibility to medical services compared to the lower educated participants [24].

In addition, this study revealed that the prevalence of undiagnosed hypertension was higher among people from the low socioeconomic status which was similar to the findings of undiagnosed hypertension in a rural district in Bangladesh [13]. The poor people have lower access to healthcare facilities in Bangladesh which may lead to have few chances to diagnose their hypertension [25]. Moreover, it is expensive for poor people to get a hypertension screening test that includes transportation time to a medical facility, long waiting time, and the monetary costs [23]. Therefore, the level of education and wealth are two of the most important socioeconomic factors for undiagnosed hypertension.

Older and female patients were likely to have lower risks of being undiagnosed for hypertension. Studies showed that in South Asian countries, the risk of cardiovascular diseases began at the age of 40 years and immunity steadily goes down with the increase in age [26, 27]. As a result, people face frequent health disorders and have to visit physicians. Nonetheless, this creates an opportunity to get diagnosed with some typical health screening, including hypertension. According to the BDHS (2011), awareness regarding hypertension was higher among the females compared to the males which undeniably leads to lower risk of being undiagnosed for hypertension [14].

It is, however, important to note that overweight individuals had a lower prevalence of undiagnosed hypertension compared to normal BMI. An obese individual possesses a higher risk of developing hypertension [28]. Hence, the chance of diagnosing hypertension is high in this group due to the higher number of physicians visits for health screening related to other chronic condition (e.g. unfavourable cardiovascular outcome, diabetes) [9, 13, 29].

The prevalence of undiagnosed hypertension varied in different health systems. Unlike high-income countries, undiagnosed hypertension is high in LMICs like Bangladesh. For example, the prevalence of undiagnosed hypertension was 18% in the USA and 17% in China [15, 24]. This is because high-income countries (e.g. USA, Australia, China, and Canada) have more functional health centres and private clinics that outfit for the needs of the citizen. Such facilities do not widely exist in LMICs [24, 30, 31].

In this study, place of residence (e.g. urban and rural residence) was not significantly associated with undiagnosed hypertension. The number of healthcare facilities (including private facilities) is higher in the urban area in comparison to the rural area [32]. However, population rate is also commensurate in the urban area compared to the rural area [33]. Moreover, access to healthcare facilities do not necessarily turn to the deployment of such facilities to expedite detection and diagnosis unless the facilities ensure proper diagnostic services for hypertension. Khanam et al. 2014 showed that qualified providers (e.g. M.B.B.S, specialized doctors) diagnosed only 53.5% and among unqualified providers, 40.7% diagnosed by the informal healthcare provider (e.g. quack, village doctors) [34]. The 2014 Bangladesh Health Facility Report suggested that the diagnostic capacity for screening tests of NCDs was low in primary level healthcare facilities of Bangladesh [35]. According to the action plan for NCDs under Health Population and Nutrition Sector Program (HPNSP), primary health care facilities can be reoriented to provide basic screening services for NCDs including hypertension [36]. However, a large number of primary level healthcare provider needs to be trained before implementing such an intervention.

BDHS 2011 reported that 66.0% of the hypertensive patients are currently taking medicine to lower the high blood pressure [14] which indicates another challenge to the health systems of Bangladesh to ensure treatment of the hypertensive patient. A recently conducted study on costing of essential service package found that on an average the cost per hypertensive patient for drugs, supplies, and human resources was 4500.5 BDT (57.5 USD) for 1 year period in primary and secondary level facilities of Bangladesh [37].

In several studies, it was evident that poor people face financial hardship while accessing healthcare in Bangladesh [38–40] due to high reliance on out-of-pocket healthcare payment. For example, Khan et al. 2017 found that the poor household faced a higher incidence of catastrophic health expenditure compared to other wealth groups [39]. In another study in rural Rajshahi, it was observed that poor economic status was one of the determinants of financial catastrophe for chronic illness [40]. Currently, healthcare services are limited at the primary level public healthcare facilities. People usually pay the high out-of-pocket payment for seeking treatment for hypertension from private healthcare facilities in Bangladesh (Masuma Akter Khanam et al., 2014; Rahman, Gilmour, Saito, Sultana, & Shibuya, 2013).

We found that residents of the Sylhet region were at lower risk of undiagnosed hypertension compared to the Khulna region. A study showed that participants from the Sylhet region were less likely to have hypertension (16.0%) compared to other regions in Bangladesh [41]. The lower prevalence of hypertension may result in a lower prevalence of undiagnosed hypertension in Sylhet region. However, this needs further examination for identifying the contextual factor of undiagnosed hypertension in Sylhet region. Currently, a limited number of studies are available on the geographical distribution of prevalence of hypertension and its risk factors in Bangladesh.

The Government of Bangladesh has documented several policies in its last Non-Communicable Disease Control Operational Plan (2017–2022). Under the Operational Plan, Primary Health Care system (Community, Community Clinic, Union Health Facility, and Upazila Health Complex) will be used for prevention of NCDs through public awareness, screening and early detection, treatment and referral. Action will be taken to develop the existing hypertension management guideline, train of doctors and non-doctors for hypertension management, smooth the supply of essential medicines and technologies, supply of subsidized treatment for poor, raise public and political awareness/understanding about NCDs and their risk factors through social marketing, mass media and responsible media reporting, and promote healthy lifestyle and practices at community level and at facility levels [42, 43]. However, there is a lack in implementing the undertaken policies in the past years [44]. In 2013, the Ministry of Health and Family Welfare has developed a guideline for detecting and treating the hypertension in Bangladesh [44]. The guideline recommended lifestyle modification and physical activity as a non-pharmaceutical treatment in early stage (stage 1) of hypertension.

The blood pressure of individuals was measured three times with 10 minutes interval between each measurement on the day of the survey rather than monitoring for a prolonged period which was a potential limitation of this study. However, multiple visits to a household for a prolonged period were not feasible for such a huge sample size. The potential risk factors identified as having the associations with undiagnosed hypertension were not causal due to the cross-sectional nature of the data. However, as the larger sample allows getting precise estimates and adjustment of confounders, we had the opportunity to find the association between the prevalence of undiagnosed hypertension and the socioeconomic factors.

## Conclusion

This study showed that a large proportion of the cases with hypertension remained undiagnosed in Bangladesh especially among the poor and the low education group. Awareness about high blood pressure is required at an individual level to check and follow-up blood pressure level routinely. Policies to prevent hypertension has already been developed in the strategic plan and operational plan, however, implementing the undertaken policies is the key issue. The primary healthcare facilities need to be strengthened by making drugs and screening facilities available for preventing hypertension. As the undiagnosed hypertension is remarkably higher besides the burden of hypertension is rising, policy focus needs to be reinforced to ensure fruitful application of it. Although publicly financed insurance and health vouchers have been proposed in the strategic investment plan to protect the poor against the cost of care for chronic diseases and catastrophic illnesses, efforts towards prevention and control of hypertension should be prioritized and planned actions under the operational plan should be properly implemented. Further, the surveillance system needs to be conducted to track the rising burden of hypertension as well as to detect the undiagnosed cases and to intervene accordingly at the community level.

## Abbreviations

AHA: American Heart Association
AOR: Adjusted odds ratios
BDHS: Bangladesh Demographic and Health Survey
BMI: Body-mass index
C: Concentration index
CI: 95% confidence intervals
DBP: Diastolic blood pressure
LMICs: Low and middle-income countries
NCDs: Non-communicable chronic diseases
OR: Odds ratios
PCA: Principal component analysis
SBP: Systolic blood pressure
SE: Standard error
